# The impact of provider payment reforms and associated care delivery models on cost and quality in cancer care: A systematic literature review

**DOI:** 10.1371/journal.pone.0214382

**Published:** 2019-04-05

**Authors:** Mina Nejati, Moaven Razavi, Iraj Harirchi, Kazem Zendehdel, Parisa Nejati

**Affiliations:** 1 The Cancer Institute at Imam Khomeini Hospital, Tehran University of Medical Sciences, Tehran, Iran; 2 The Schneider Institutes for Health Policy at the Heller School of Brandeis University, Waltham, MA, United States of America; 3 Rasoule-Akram Hospital, Iran University of Medical Sciences, Tehran, Iran; RTI International Metals Inc, UNITED STATES

## Abstract

**Objectives:**

To investigate the impact of provider payment reforms and associated care delivery models on cost and quality in cancer care.

**Methods:**

**Data sources/study setting:** Review of English-language literature published in PubMed, Embase and Cochrane library (2007–2019).

**Study design:** We performed a systematic literature review (SLR) to identify the impact of cancer care reforms. Primary endpoints were resource use, cost, quality of care, and clinical outcomes.

**Data collection/extraction methods:** For each study, we extracted and categorized comparative data on the impact of policy reforms. Given the heterogeneity in patients, interventions and outcome measures, we did a qualitative synthesis rather than a meta-analysis.

**Results:**

Of the 26 included studies, seven evaluations were in fact qualified as quasi experimental designs in retrospect. Alternative payment models were significantly associated with reduction in resource use and cost in cancer care. Across the seventeen studies reporting data on the implicit payment reforms through care coordination, the adoption of clinical pathways was found effective in reduction of unnecessary use of low value services and associated costs. The estimates of all measures in ACO models varied considerably across participating providers, and our review found a rather mixed impact on cancer care outcomes.

**Conclusion:**

The findings suggest promising improvement in resource utilization and cost control after transition to prospective payment models, but, further primary research is needed to apply robust measures of performance and quality to better ensure that providers are delivering high-value care to their patients, while reducing the cost of care.

## Introduction

Cancer care is moving toward more advanced, targeted treatments that have the potential to improve health outcomes. Despite the substantial progress in economic evaluations of medical interventions, policy makers are increasingly challenged to control rapidly rising healthcare costs. Available projections for cancer care cost in the United Kingdom, the United States (US), and Australia suggest a dramatic increase from 42% to 66% above their current levels by 2025 [1], [2],[3]. During the past five years, 68 high-cost, novel therapies were launched globally for the treatment of cancer [4], so now the challenge is developing more compelling evidence on whether this expensive paradigm shift leads to better outcomes. Additional increases in cancer treatment costs are associated with the availability of these high-cost novel agents, as well as use of combination therapies and multiple lines of treatment [4]. As treatment options increase, further efforts are required to ensure that providers apply efficient resource-utilization strategies without adversely affecting the quality of care.

Multiple policy reforms have been launched in cancer care, but provider payment models remain an area of contention among policy makers. Shifting the orientation of the provider reimbursement system from services to outcomes requires the identification and reward of high-quality, patient-centered care [5–9]. This transition is tied to care coordination and service delivery models to establish the components of a value-based system. Therefore, a wholesale change in practice is expected to take place while adapting performance metrics, operation systems, and legislative structures to provide value to patients [6, 9–11].

Although the interaction between payers and providers suggests a move from a volume-based to value-based model in oncology practice, there is still limited evidence on successful payment reforms. We conducted a systematic literature review (SLR) to identify and summarize existing evidence on the impact of provider payment reforms and care delivery models on cost and quality measures in cancer care. Previously published literature reviews on this topic were conducted on the basis of existing narrative reviews, expert opinions, and evaluation research studies [12–14]. However, this is the first SLR that focuses on methodological studies reporting comparative data on cost, quality, and clinical outcomes to first describe the landscape of policy reforms targeting inefficiency of care, and second to fill the existing knowledge gap on the effectiveness of such experiments in providing value to patients diagnosed with cancer.

## Materials and method

### Data sources and searches

The SLR was conducted in accordance with the Preferred Reporting Items for Systematic Reviews and Meta-Analyses (PRISMA) guidelines to identify English-language articles published between January 1, 2007, and January 15, 2019, reporting the impact of provider payment reforms and care delivery models in cancer care. Searches were conducted in PubMed, Embase, and the Cochrane Library; S1, S2 and S3 Tables present the search algorithms for each database. This search was supplemented by manual bibliography searches of the identified studies and authors.

### Study selection and data extraction

**Fig 1:**
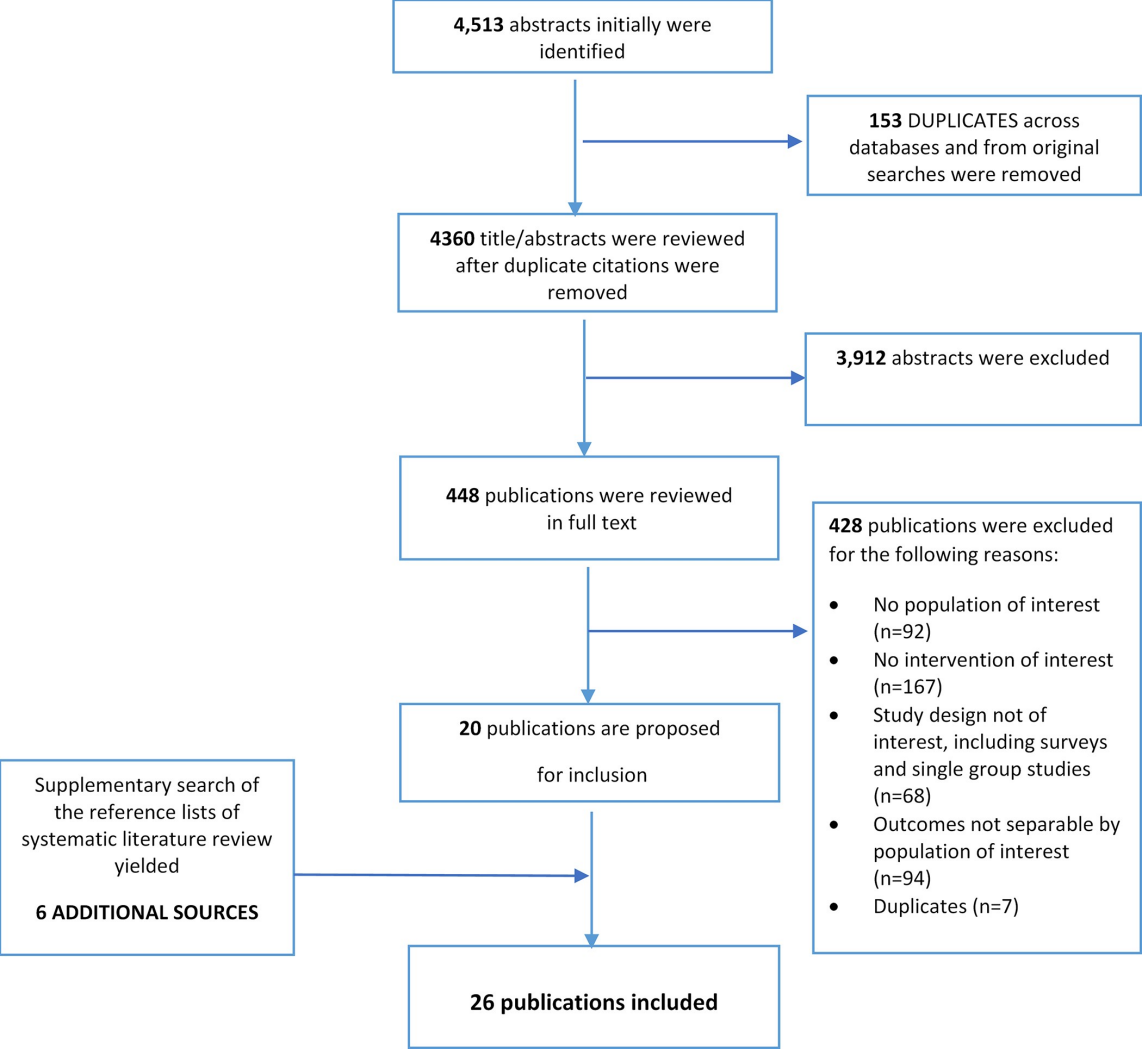
Overview of the systematic literature search: PRISMA flow chart. Using a standardized data extraction form, the mean change and standard deviation of outcome measures within the intervention and comparator groups were extracted and validated accordingly for additional quality assurance. Studies published in multiple articles were extracted as one study. A risk-of-bias assessment for included publications was undertaken by independent reviewers using a checklist developed by the Cochrane Collaboration, the Risk of Bias in Non-Randomized Studies of Interventions (ROBINS-I) tool for non-interventional studies [15]. The seven domains used in the ROBINS-I tool determine the strength of evidence/risk of bias due to confounders, participant selection, classification of interventions, deviations from intended interventions, missing data, measurement of outcomes, and selection of the reported results. A three-point Likert scale was applied to score the bias from low to serious for each of the seven domains; this was later validated by a second reviewer.

As show in the Patients, Interventions, Comparators, Outcomes, Time and Study design (PICOT) selection criteria table (Table 1). primary endpoints were healthcare resource use, quality, cost, and clinical outcomes. Eligible studies were required to report the impact of policy reforms comparatively in any cancer care setting. The impact was defined as consequences of identified interventions on any of these outcome measures. Interventions related to care delivery models were eligible if they were linked to the provider payments either directly or implicitly. Case reports of policy reforms were eligible if research methodology was clarified and met the inclusion criteria. Commentaries, editorials, narrative reviews, and genetic studies, as well as those with no separable outcomes for cancer care were excluded. Two rounds of screening took place to identify the relevant literature. In the first round, each title/abstract was reviewed by one reviewer for inclusion using the PICOT criteria. A 10% random sample of the articles rejected during this phase were validated by a second reviewer. In the second round of screening, the full texts of the articles identified during the first pass were reviewed and validated (see the flow of literature presented in the PRISMA diagram in Fig 1).

**Table 1:** PICOT inclusion and exclusion criteria for study selection in the SLR.

Criteria	Inclusion criteria	Exclusion Criteria
Population	• Patients diagnosed with cancer • Physicians providing cancer care either in the outpatient or inpatient setting • Payers involved in cancer care reimbursement	Publications that do not evaluate cancer care.
Intervention Comparator	**Explicit payment reforms directly involving financial incentives:** Value-based payment, bundled payments, shared savings programs, partial or full capitation, fee-for-service (FFS), global budget, pay-for-performance (P4P), Medicare Modernization Act, other oncology-specific reimbursement models **Implicit payment reforms through care coordination:** Accountable care organizations (ACOs), oncology care model (OCM), patient-centred oncology medical home (PCMH), oncology pathways, Other cancer care delivery models under the umbrella of payment reforms	Studies that do not report the interventions of interest in cancer care Local or small-size initiatives associated to the care delivery reforms including hiring patient navigators or implementing triage phone policies
Outcomes	• Utilization / healthcare resource use: hospitalization, physician visits, outpatient visits, ICU admissions, Emergency department visits, Specialist visits, length of stay, chemotherapy medication use, time on treatment • Direct and indirect costs of care: hospital costs, total costs, medication costs • Quality of care, adherence to standard of care • Clinical outcomes: survival rate, response to treatment and cancer related mortality	Studies that do not report outcomes of interest for the study population or outcomes not reported separately for cancer care.
Study design	Economic analyses, all epidemiological and observational study designs including but not limited to prospective or retrospective cohort studies, cost-of-illness studies, Database/claims data analyses, case reports on policy reforms	Animal, in vitro, or genetic studies, comments/commentary, news, editorials, or narrative reviews
Other limits	limited to articles with an abstract published in English since January 1, 2007 to Jan 15, 2019	Studies published prior to 2007 or after the final search date in 2019, or not published in English

## Results

**Fig 2:**
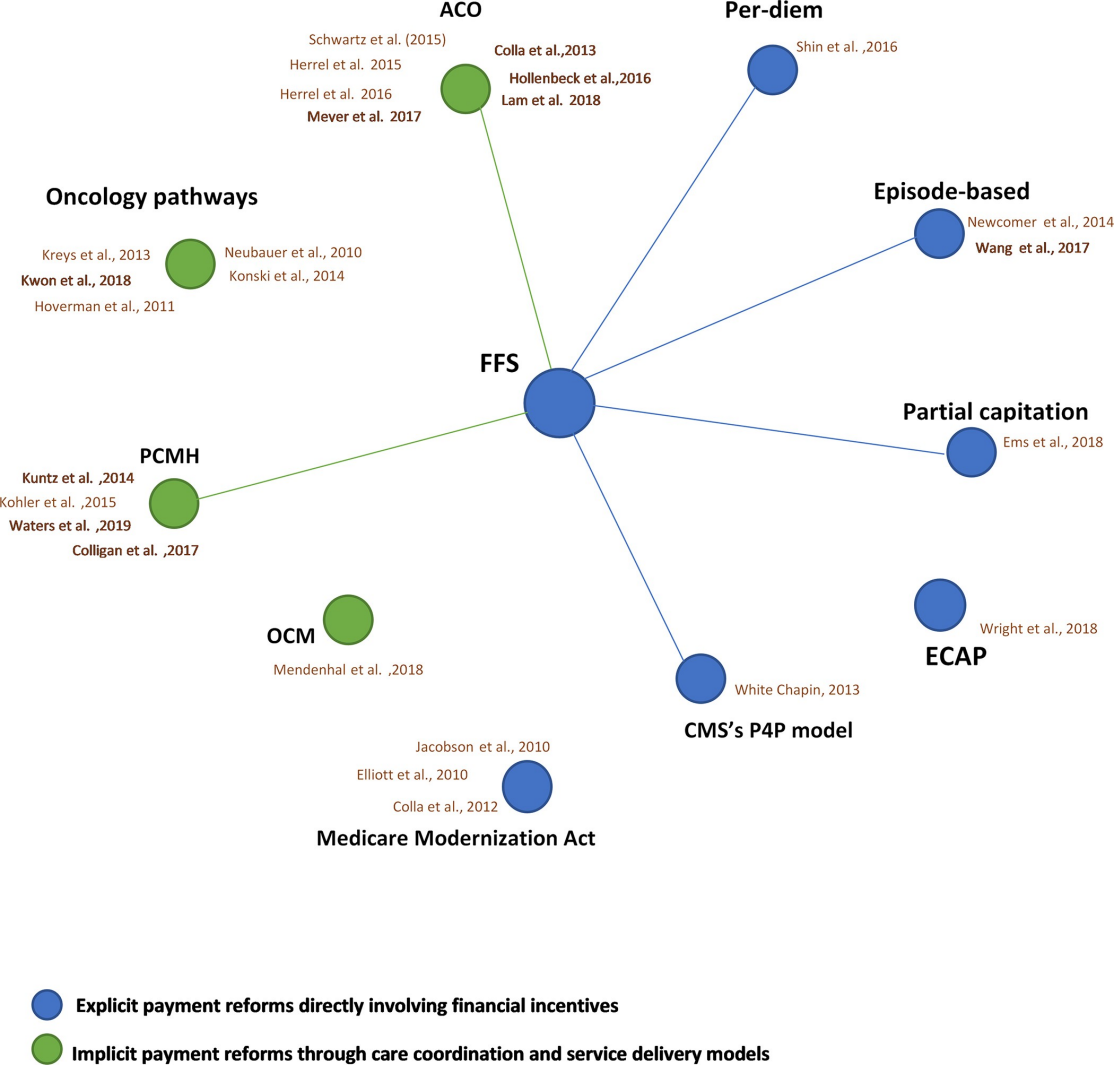
Evidence network for the provider payment reforms in cancer care identified in the SLR. Direct line indicates head-to-head comparison of the interventions as emerged from the data. ACO, accountable care organization; CMS, centers for Medicare and Medicaid services; ECAP, endometrial cancer alternative payment; FFS, fee-for-service; P4P, pay for performance; OCM, oncology care model; PCMH, patient centered medical home.

The database and hand-searches yielded 4,513 articles, 26 of which were included in the SLR. Fig 2 presents the network of evidence identified in the literature, providing a clear picture of the models that have been carried out in cancer care. Any studies that were conducted in non-cancer settings (n = 58) or reported no separable outcomes for cancer care (n = 13) were excluded from the SLR. Most of the studies were conducted in the US, except for two in South Korea and one in Taiwan.

The included studies presented limited data on the definition of outcomes used, or similar outcomes were measured in different ways. Given the observed variation in study populations, interventions, and outcome measures, we found it inappropriate to perform a meta-analysis to estimate the effect size of each identified model. Taking statistical considerations into account, we used thematic analysis to generate descriptive and explanatory themes as they emerged from the data. Key themes were organized according to the type of reform. All reimbursement models directly involving financial incentives to providers were grouped as explicit payment models. Implicit payment reforms carried out following care-coordination programs, delivery models, or quality initiatives were categorized as implicit payment models.

Using the ROBINS-I tool, we found potential sources of bias in these studies. Of the seven domains scored, confounding and selection bias were found to be the main drivers of risk of bias, followed by outcome measurement and reporting the results. This is perhaps due to the nature of the included studies, with no to very limited control on confounders and outcome measurements. In total, findings indicated that the outcomes reported were moderately biased across the studies.

### Explicit provider payment methods

The impact of financial incentives to cancer care providers was investigated in the context of the following models. The findings are summarized in Table 2.

**Table 2:** Summary of the studies reporting data on the impact of implicit payment reforms in oncology practice.

Author, year	Geographic location	Payment Method; Intervention vs. comparator	Study design Data source	Cancer type Sample size	Outcomes measures	Findings
Newcomer et al. (2014)	US	Episode based payment vs. predicted costs in FFS setting	Retrospective cohort study using FFS-based registry data	Colon, lung and breast cancers Sample size.N = 810	• Total treatment costs • Chemotherapy drug cost • Overall Survival	**Episode-based payment vs. FFS:** • Actual treatment costs: -34% • Chemotherapy drug costs (CDC): +179% • No significant impact on patients’ survival and quality of care (subbgroup of lung cancer survivors)
White et al. (2015)	US	Care management and performance-based payment (P4P) vs. FFS	Simulation model developed by centers for Medicare and Medicaid services (CMS) using Medicare claims data from the chronic condition warehouse (CCW)	Eight cancer types.Sample size N = 330,647 episodes	Changes in total spending under three scenarios of behavioral responses to the P4P reform	**Total spending in P4P vs. FFS model:** • Under the “no behavior” scenario: 4% increase • Under the “%5 reduction” scenario: 1% decrease • Scenario of “10% reduction”: 5% reduction
Shin et al. (2017)	South Korea	Per-diem Payment System (PDPS) vs. FFS	Quasi experimental model using claims data (Difference in difference method)	More than twelve cancer types.Sample size.N = 5464	• Length of stays • Total medical costs • Treatment costs • Medications cost • Cost of laboratory and radiological tests	**PDPS vs. FFS-based hospitals:** • Length of stays per episode: -2.56%, p = 0.0001 • Total medical costs: -2.46%, p = NR • Duration per episode: -2.5% (95% CI: -0.0324, -0.0194), p<0.0001 • Treatment costs per day: -4.4% (95% CI: -0.071, -0.018), p<0.0001 • Analgesic medications cost per day: -0.59% (95% CI: -0.007, -0.0047), p<0.0001 • Cost of lab tests: -6.62% (95% CI: -0.0688, -0.0683), p<0.0001 **PDPS (Post-Implementation vs. baseline):** • Total medical costs: -0.76%, p = NR
Elliott et al. (2010)	US	Physician reimbursement reform in the Medicare Modernization Act	Retrospective cohort study using surveillance, epidemiology, and end results database (SEER)and Medicare claims data	Prostate cancer.Sample size.N = 72,818	Utilization of androgen suppression therapy (AST) at first year	**Post vs. pre-reform:** • Non-indicated AST use in low risk patients: -39%, p<0.001 • No significant change in indicated AST among low risk patients • No significant change in AST use among metastatic patients
Ems et al. (2018)	US	Partial capitated-payment model (post-vs. pre) vs. FFS method	Quasi experimental model using data from a Medicare Advantage plan	Mix of cancer types (Not specified).Sample size.N = 713	• Chemotherapy-related Complications • Ambulance transports	**Post- vs. pre-contract capitated:** • Significant Increase in mean number of chemotherapy-related complications (p = 0.01) and ambulance transports (p<0.0001) Postcontract vs. Precontract FFS: • No difference in any outcome measure
Jacobson et al. (2010)	US	Physician reimbursement reform in the Medicare Modernization Act	Retrospective cohort study using data from SEER registry	Lung cancer.Sample size: not reported	• Chemotherapy use • Change in treatment choices	**Post vs. pre-reform:** • Chemotherapy prescriptions within 1-month: +2.4%, p<0.001 • Increase in prescription rate of more expensive drugs (Docetaxel: 13%-20% increase)
Colla at al. (2012)	US	Physician reimbursement reform for end of life chemotherapy in the Medicare Modernization Act	Retrospective cohort study using Medicare claims data	Mix of cancer types (Not specified).Sample size: not reported	Chemotherapy use at the last 14 days, 3- month or 6-month of life	**Post vs. pre-reform:** • The last 14 days of life (visited in physician offices) = -20% (95% CI: -4.2 to -1.0, P = 0.002) • The last 14 days of life (hospital outpatients): no changes (95% CI: -2.4 to 4.4; P = 0.541). • last 3 and 6 months of life (any practice setting): Significant reduction in number of treatments per patient p<0.001.
Wang et al. (2017)	Taiwan	Episode-based (bundled-payment) vs. FFS	Retrospective cohort study with a matched control group using national registry and claims data	Breast cancer.Sample size.N = 17,940	• Adherence • 5-year event-free survival rate • 5-year medical payments	**Bundled payment vs. FFS:** • Greater adherence to quality indicators (p<0.001) • Greater 5-year event-free survival (p<0.01) • Stable 5-year medical payments in bundled payment model vs. 20% increase in FFS arrangement
Wright et al. (2018)	US	Endometrial cancer (EC) alternative payment (ECAP) model	A decision model used data from MarketScan and Medicare	Endometrial cancer (EC).Sample size.N = 29,558	Potential cost savings through lower case reimbursement	**In the optimized care model:** Reduction in average case reimbursement in both public (-2.9%) and commercial payers (-5.9%)

#### ■ Per-diem vs. FFS

A single study examined the transition from fee-for-service (FFS) reimbursement to a per-diem payment system (PDPS). This study involved patients from seven hospitals in a PDPS program with FFS-based centers [16]. Compared to the controls in the FFS setting, per-diem reimbursement significantly reduced length of hospital stays (p = 0.0001) and total medical costs for PDPS participants. Besides a 2.5% decrease in duration of care (p<0.0001), costs associated with lab tests, cancer treatment, and analgesic medications dropped by 6.62%, 4.4%, and 0.59%, respectively (p<0.0001). The average reduction in length of hospital stays was 3.19 days post implementation of the per-diem model [16].

#### ■ Episode-based payment vs. FFS

The impact of episode-based reimbursement in cancer care was investigated in two studies, conducted in Taiwan [17] and the US [18], where significant improvements in the five-year cost and quality outcomes were reported compared to the regular FFS payment. A large cohort of Tai patients with stage 0-III breast cancer (n = 17940) in the bundled-payment program were matched with controls in an FFS setting; the results indicated significantly better adherence to quality indicators (p<0.001) and an improved five-year survival rate (p<0.01) within the bundled-payment system. Although the cumulative medical payments for the FFS group increased by 20%, the five-year medical payments of the bundled-payment group remained stable[17]. Similarly, in the US-based healthcare system, direct costs of cancer care in an episode-based program dropped by 34% compared to the predicted costs in a large FFS-based registry, resulting in a net savings of $33,361,272 [18]. However, drug costs increased by 179%, with a net rise of $13,459,913 due to the change in chemotherapy prescriptions. A subset analysis of 50 medical groups with at least 70 participants per episode demonstrated the same cost impacts as the base-case analysis. When using data from another subgroup of lung cancer survivors, no significant difference was found in patients’ survival between the two payment groups.

#### ■ Pay-for-Performance (P4P) vs. FFS

A simulation model investigated the impact of a performance-based payment on specialty oncology services compared to FFS spending [19]. Using historical episodes from Medicare claims for FFS beneficiaries, the Centers for Medicare and Medicaid (CMS) established a model for prevalent cancers (n = 330,647 oncology episodes). Assuming a target spending for chemotherapy episodes, an extra performance-based compensation per episode was paid to physicians in addition to the standard FFS-based payment in practices with episode costs falling below the target. Changes in the quantity and intensity of services in response to the P4P reform were measured under one of three scenarios: “no change”, “5% reduction” and “10% reduction” from the initial FFS spending. The study found a range of -5% to +4% change in total spending under the “10% reduction” and “no behavior” scenarios, respectively, indicating a substantial drop in total costs after implementing the P4P model [19].

#### ■ Capitated-payment model vs. FFS

We identified a single study reporting the impact of an oncology group’s transition from FFS arrangement to a partial-capitated model [20]. A six-month follow-up of Medical Advantage enrollees from a single health plan in the US indicated that there were no significant differences in all-cause hospitalizations between the two payment cohorts. Although the mean number of chemotherapy-related complications and ambulance transports were greater post-transition, the subset analysis indicated that the post-capitated group was sicker than the pre-capitated cohort (according to the Deyo-Charlson Comorbidity Index (CCI) and the Rx-Risk scores).

#### ■ Other explicit payment reforms

Three studies reported the impact of provider payment reform implemented as part of the Medicare Modernization Act, and determined it was effective in switching treatment patterns and chemotherapy use [21–23]. According to the findings, regardless of the practice setting, dropping reimbursement rates significantly reduced chemotherapy prescriptions per patient at three and six last months of life for patients diagnosed with a variety of cancer types (p<0.001)[21]. Similarly, a major reduction (64%) in reimbursement for androgen suppression therapy (AST) in prostate cancer (n = 72,818) significantly reduced the odds of receiving non-indicated AST (given without other therapies such as radical prostatectomy or radiation) in low-risk patients (p<0.001) [22]. In contrast, in a cohort of lung cancer patients, outpatient chemotherapy prescriptions significantly increased, with the pattern of switching to high-margin drugs because of the reduction in reimbursement rates. A 2.4% increase was observed in chemotherapy use less than one month after reducing payment rates for lung cancer services (p<0.001)[23].In one study, the endometrial cancer alternative payment (ECAP) model, a value-based healthcare reform initiated based on the Physician Payment Reform Taskforce (PPRTF) in the Society of Gynecologic Oncology (SGO), was investigated for sources of potential cost savings while promoting quality of care[24]. An optimized care model was created using claims data from MarketScan and Medicare databases. The model focused on the rate of minimally invasive surgery, length of stays, and emergency department (ED) visits/readmissions to compare mean reimbursement per episode of surgical care among low-risk, early-stage EC patients (n = 29,935). Outcomes were stratified by three surgical approaches, from 30 days preoperatively to 60 days postoperatively. The model derived a cost savings of $903 per patient (2.9% of baseline) in MarketScan, and $1243 (5.9% of baseline) in Medicare, as reflected in the proposed value-based reimbursement strategy.

### Implicit payment reforms targeting delivery of care

Eleven studies in the review reported data on care delivery reforms, including adoption of clinical pathways and participation in patient-centered medical home (PCMH), accountable care organization (ACO), or oncology care model (OCM) programs. The findings are synthesized and summarized by type of reform within the following sub-sections and Table 3.

**Table 3:** Summary of the studies reporting data on implicit payment reforms through care coordination in oncology practice.

Author, year	Country	Cancer care delivery models;.Intervention vs. comparator	Study design.Data source	Cancer type.Setting.Sample size	Outcomes measures	Findings
Hoverman et al. (2011)	US	Adopting level-I colon cancer pathways vs. off pathway treatments	Retrospective cohort study using data form US Oncology Network and MedStat databases	Colon cancer.Sample size.N = 1130 patients	• Total costs per case • Total costs per patient per month • Chemotherapy cost • Chemotherapy duration • Chemotherapy related admissions • Overall survival (OS) • Disease-free Survival (DFS)	**On vs. off pathway treatment in adjuvant group** • Total costs per case: -35%, p<0.001 • Treatment costs per patient: -35%, p≤0.01 • Chemotherapy costs: -63%, p<0.001 • Chemotherapy period: -34%, p<0.001 • Chemotherapy admissions: -23%, p = 0.236 • DFS: HR = 4.98 (95% CI, 2.11 to 11.74), P<0 .05 In metastatic patients only OS was significantly longer: 26.9 vs. 20.1 months,P = 0.03
Konski et al. (2014)	US	Pathway-based vs. off-pathway hypofractionation	Simulation Model using a sample hospital-based data from a radiationoncology practice	Breast, prostate and lung cancers.Sample size.N = 221	• Per-patient revenue • Changes in technical workflow • Professional relative value unit per case (RVU)	**On vs. off-pathway treatment:** • Annual reduction in global revenue: $540,661 • Workflow reduction: five patients or 1 to 1.5 operating hours per day RVU reduction: -2,121
Kreys et al. (2013)	US	Before vs. after adopting clinical pathways	Retrospective cohort study using claims data from CareFirst BlueCross BlueShield of Maryland	Breast, colorectal, lung.Sample size.N = 4,713 (across 46 cancer centers)	• Hospitalization costs • Drug costs • Hospitalization days • Number of hospitalizations • Total cost savings	**Two-year after vs. 1-year before adoption:** • hospitalization costs per patient: -75%, p = 0 .004 • Number of hospitalizations per patient (30 days post-chemotherapy): -8.7%, p = 0.255 • Hospitalization days per patient: -32.8%, p = 0.057 • Total drug costs per patient: +2.5%, p = 0.587 • Total cost savings compared to the projected costs: $10.3 million
Kwon et al. (2018)	South Korea	Pathway-based vs. off-pathway treatment of thyroid cancer	Retrospective cohort study	Thyroid cancer.Sample size.N = 361	• Length of hospital stays • Total costs per patient	**On vs. off-pathway treatment:** Length of hospital stays: -0.8 days (p = 0.02) Cost per patient: -14.8% (p<0.001)
Neubauer et al. (2010)	US	Before vs. after adopting level I clinical pathways for the treatment of lung cancer	Retrospective cohort study using data form US Oncology Network	Non-small cell lung cancer.Sample size.N = 1,409 patients across eight cancer centers	12-month costs • Outpatient costs • Cost of medications • OS	**On vs. off-pathway treatment:** • Outpatient costs: -35% • Cost of chemotherapy: -37% • Non-chemotherapy medications: -39% • 12-month OS: +2.2%, p = 0.867
Kohler et al. (2015)	US	PCMH vs. non-PCMH arrangement	Retrospective cohort study using North Carolina Medicaid claims	Breast cancer Sample size N = 758 (9407 person-month observations)	• Outpatient visits • Inpatient hospitalizations • Emergency Department (ED) visits • Medicaid expenditures	**PCMH vs. non-PCMH enrollees:** • Monthly Medicaid expenditures within the first 15-month from diagnosis: -22.9%, p<0.001 • Outpatient visits: -22.2%, p<0.001 • Inpatient hospitalizations: no differences, p = NR • ED visits: no differences, p = NR
Kuntz et al. (2014)	US	Michigan Oncology Medical Home Demonstration Project at first year vs. 3-year data from a historical control group	Retrospective cohort study using historical data	Mix of cancer types (Not specified).Sample size.N = 519	• ED visits • Inpatient admissions	**After vs. before implementing the model:** • ED visits per patient: -47% Inpatient admissions per patient: -68.2% • Cost savings per patient: $550
Waters et al. (2019)	US	Community Oncology Medical Home program (COME HOME) vs. FFS	Quasi experimental model using claims data (Difference in difference method)	Seven cancer types.Sample size.N = not reported (across seven cancer centers)	• Medical spending • ED visits • Ambulatory care • Hospitalizations • Readmission rate • Length of stay	**Medical home vs. FFS:** • Change in 6-month medical spending post implementation: -$2,657, p = 0.008 • ED visits per 1,000 patients: -10.2%, p = 0.024 • No significant change was observed in other outcome measures
Colligan at al. (2017)	US	Community Oncology Medical Home program (COME HOME) vs. FFS	Retrospective study using matched control group from Medicare claims	Seven cancer types.Sample size.N = not reported (across seven cancer centers)	Average costs over the last 30, 90 and 180 days of life	Medical home vs. FFS • Last 30-day costs: -$959 (-6%) • Last 90-day costs: -$3,346 • Last 180-day costs: -$5,790
Colla et al. (2013)	US	The Physician Group Practice Demonstration project for ACO participants vs. non-ACO	Quasi experimental model using Medicare claims (Difference in difference method)	Mix of cancer types (Not specified).Sample size.N = 988,781 person-years	Annual per-beneficiary change in payments	**ACO vs. non-ACO enrollees post-reform:** Annual per-beneficiary change in payments: -3.9%
Herrel et al. (2015)	US	ACO participating hospitals vs. non-ACO hospitals	Retrospective cohort study using national inpatient sample	Patients with urologic cancer who underwent elective major surgery Sample size: • Non-ACO hospitals: n = 352 • ACO hospitals: n = 176	• In-hospital mortality Prolonged length of stay (LOS, >90^th^ percentile) • Total hospital costs	**ACO vs. non-ACO hospitals:** • Mortality rate: no significant difference, p = NR • Length of stay: no significant difference, p = NR • Total inpatient costs: no significant impact on prostatectomy (p = 0.07), nephrectomy (p = 0.07) and cystectomy (p = 0.25) services
Herrel et al. (2016)	US	ACO vs. non-ACO practices	Quasi experimental model using Medicare claims (Difference in difference method)	Nine cancer types Sample size: 384,519 patients across: • ACO hospitals: 106 • Non-ACO hospitals: 2,561	• 30-day mortality rate • Complication rate • Readmission rate • Length of stay	**ACO vs. non-ACO hospitals**: • 30-day mortality rate: 3.3% vs. 3.4%, p = 0.54 • 30-day readmission rate:12.5% vs. 12.4%, p = 0.69 • Complications rate: 43.6% vs. 43.4%, p = 0.65 Length of stays: 10.1% vs. 10.2%, p = 0.56
Hollenbeck et al. (2017)	US	ACO vs. non-ACO practices	Retrospective cohort study using Medicare claims	Prostate cancer.Sample size.N = 15,640	• Use of curative treatment • Total spending	**ACO vs. non-ACO enrollees** • Use of curative treatment: no significant change, p = 0.33 Spending one-year after diagnosis: +5.8%, p = 0.03
Lam et al. (2018)	US	ACO vs. non-ACO practices	Quasi experimental model using Medicare claims (Difference in difference method)	Eleven cancer types.Sample size.N = 622,080	• Total spending • Inpatient spending • Outpatient spending • Physician services • Chemotheray spending	**ACO vs. non-ACO enrollees post-reform:** • Total spending: no significant change, p = 0.94 • No significant change in inpatient (p = 0.31), outpatient (p = 0.32), physician services (p = 0.81), and chemotherapy spending (p = 0.81)
Meyer et al. (2017)	US	ACO vs. non-ACO practices	Retrospective cohort study with a matched control group using Medicare claims	Breast and prostate cancers.Sample size.N = 1,480,414	The prevalence of breast and prostate cancer screening	**ACO vs. non-ACO enrollees** • The prevalence of breast cancer screening: +40%, p<0.001 • The prevalence of prostate cancer screening: +31.7%, p<0.001
Schwartz et al. (2015)	US	ACO vs. non-ACO practices	Quasi experimental model using claims data (Difference in difference method) using Medicare data	Mix of cancer types (Not specified).Sample size.N = 17516641	Reduction in Low-value service use	**ACO vs. non-ACO hospitals:** • Utilization of cancer screening/imaging services per 100 beneficiaries: -2.4% (95% CI: -4.1, -0.7). • Total spending on low-value services: -4.5% (p = 0.004).
Mendenhal et al. (2018)	US	Oncology Care Model (OCM) program	Retrospective cohort study using data from Chronic Condition Warehouse	Mix of cancer types (Not specified).Sample size.N = 1,600	• Acute care admissions • Inpatient costs	**OCM vs. non-OCM participants:** • Acute care admissions rate: -16%, p = 0.005 • Net savings in inpatient costs in the first year: $798,000

#### ■ Adopting clinical pathways

The adoption of clinical pathways was consistently associated with reductions in resource use and costs for a variety of cancer types. Pathway-based treatment of patients with thyroid cancer (n = 361) was significantly effective in reducing length of hospital stays (p = 0.02) and cost per patient (p<0.001) compared to the controls with off-pathway treatment [25]. Likewise, adopting a clinical pathway program across 46 cancer settings (N = 4,713) was associated with a 75% reduction in hospitalization costs per patient over three years (p = 0 .004), as well as a 30% drop in cost of supportive care per patient (p<0.001). Chemotherapy drugs and hospitalization costs were found to be the main drivers of these trends, corresponding to 68% and 32% of the total savings, respectively [26]. The same impact was observed for the pathway-based treatment of colon cancer, with a substantial reduction in total adjuvant treatment costs for non-metastatic disease, and a longer survival time for metastatic patients, compared to the off-pathway group [27]. Significant reductions were also found in mean total costs per case (p<0.001), treatment costs per patient per month (p≤0.01), chemotherapy costs (p<0.001), and chemotherapy period (p<0.001) for on- versus off-pathway treatment [27]. A 35% reduction in outpatient costs was observed for patients with non-small-cell lung cancer (NSCLC); however, no significant difference was found in 12-month survival of such patients treated in the on-pathway versus off-pathway groups (p = 0.867)[28]. Regarding the impact on resource use and hospital-based billing, evidence-based hypofractionation was found to be effective in reducing five patients per day equivalent to one to 1.5 daily operating hours in a radiotherapy department [29].

#### ■ PCMH vs. FFS

The impact of the Community Oncology Medical Home program on cancer care spending was compared with FFS Medicare beneficiaries diagnosed with various cancer types. After adjustment for clinical and sociodemographic differences, implementing PCMH model was found to be associated with significantly reduced (10.2%) ED visits per 1,000 patients (p = 0.024), resulting in 8.1% savings relative to average spending per beneficiary over six months [30]. A similar result was observed for Medicare expenditures in the last year of life for beneficiaries with cancer. The average end-of-life costs in the last 30, 90, and 180 days dropped for patients in a PCMH program compared to the FFS Medicare claims for similar outpatient oncology practices [31]. Michigan Oncology Medical Home was also effective in reducing the first-year costs through avoiding unnecessary ED visits and inpatient admissions across four cancer practices, with an average estimated cost savings of $550 per patient [32]. However, according to another study, the impact of PCMH enrollment was, in some measure, associated with increased resource use and cost. A significant increase in monthly Medicaid expenditures and the number of outpatient visits for PCMH enrollees was observed over the first 15 months from diagnosis compared to non-PCMH participants (p<0.001), with no significant impact on inpatient hospitalizations or ED visits [33].

#### ■ ACO vs. non-ACO (FFS)

Compared to the non-ACO participants, Medicare beneficiaries with prostate cancer who were aligned with ACOs (n = 1,100) had similar rates of treatment (p = 0.33), but significantly increased spending in the year after diagnosis [34]. In another study on patients with urologic cancer who underwent elective major surgery, no significant differences were observed in total inpatient costs, length of hospital stays, and mortality rate among patients within 176 ACO hospitals compared to 352 non-ACO sites [35]. Similarly, the ACO model was found to have no significant association with post-surgery complication rate (p = 0.65), prolonged length of stays (p = 0.56), and readmission rate (p = 0.69) among patients undergoing major resection surgeries (n = 384,519) in ACO hospitals, compared to the non-ACO enrollees within 2,516 cancer centers [36]. In a larger scope, across 11 different cancer types from five-year national Medicare claims, having a cancer diagnosis in a Medicare ACO had no significant impact on healthcare spending and resource utilization [37]. However, in specific medical scenarios, the first year of the Medicare Pioneer ACO program was found to be effective in reducing the utilization of low-value services, defined as providing minimal or no average clinical benefit. Compared to the control group, a reduction of 0.8 services per 100 beneficiaries was observed in the ACO model with 693,218 person-years. This corresponds to a 4.5% drop in total spending on low-value services (p = 0.004). The greatest absolute reductions in service utilization post-ACO participation was observed in cancer screening and imaging tests, the most frequently delivered services [38].

Similarly, in a study by Colla et.al [39],the Physician Group Practice Demonstration ([PGPD] used as a proxy for ACO) was significantly associated with an annual reduction of 3.9% in payments per beneficiary. Major reductions in inpatient service use, hospital discharges, and intensive care unit (ICU) stays were identified, but no changes in cancer-specific procedures or chemotherapy were observed post-PGPD program. In cancer preventive care, ACO enrollment was also effective in increasing the utilization of preventive screening tests among patients with breast or prostate cancer. The prevalence of screening tests for both cancers was higher among ACO participants compared to non-ACO enrollees (p<0.001) [40]. Although the screening for prostate cancer was in the ACO model, it was not recommended by the US Preventive Services Task Force (USPSTF). Thus, delivering high-quality care in ACO models needs to be linked to the standard of care in order to control unnecessary use of low-value services.

#### ■ OCM

The SLR identified a cancer care delivery reform, implemented as a five-year model by CMS to provide coordinated, high-quality care for oncology practices using alternative payment arrangements with CMS; this included financial and clinical performance accountability for episodes of care. Mendenhall et.al [41] used risk-adjusted national averages of all practices providing cancer care in the same patient risk quartile as the comparator cohort to evaluate the impact of a multifaceted care delivery approach targeting unnecessary ED and hospital admissions. The strategy included increased care coordination, adopting standardized pathways, urgent care tactics, and patient education. The findings indicated that there was a statistically significant reduction (16%) in hospital admissions (p = 0.005), resulting in a net saving of $798,000 in inpatient costs per quarter for 1,600 patients in the first year of the OCM program. Additionally, quarterly basis surveys demonstrated that OCM patient satisfaction scores improved over the course of year 1 in the program.

## Discussion

Payment reform requires more compelling evidence to ensure providers are delivering high-value, quality care to their patients, while reducing the cost of such care. An SLR such as this can help stakeholders and payers understand the successes and failures of the reforms. Our review found that alternative payment models demonstrated promising outcomes in reducing healthcare resource use and costs, although they are still lacking robust measures of performance and quality. Of the interventions involving reimbursement reforms either directly or implicitly through care coordination models, most improved resource utilization and/or cost of care. Consistent with the previously published literature [42–45], adopting oncology pathways significantly reduced chemotherapy duration, hospitalizations, length of stays, and associated costs by avoiding unnecessary use of low-value services. The common method used to determine the effectiveness of pathways was evaluating whether their use resulted in cost savings relative to off-pathway treatments across all studies. However, this savings could not be linked to the cost drivers, which vary across cancer types and mainly depend on patient characteristics, disease stage, goals of therapy, available treatment options, and patient preferences. Although standardization of care appeared to be an effective strategy in targeting expensive chemotherapy treatments, little information was available regarding the impact of adherence to guidelines on clinical outcomes, treatment complications, and patient’s quality of life and satisfaction. It is unlikely to find a randomized trial with off-pathway versus pathway-based treatment arms; therefore, the strength of retrospective evaluation is in the ability to examine comprehensive electronic health records and claims data. The impact of adherence to standards can be more definitively measured as pathways are further adopted and implemented in cancer care, allowing for assessments of different modalities in a variety of settings in the short versus long term.

This review also identified notable findings regarding other implicit payment models that involve care coordination across providers. With the PCMH model, most studies reported potential savings in costs of care derived from significant drops in ED visits and inpatient admissions; in contrast, longer follow-up of patients until 15 months post-diagnosis indicated significant increase in outpatient service use and average expenditures per month. The increase in outpatient services for breast cancer is likely due to the greater access to care through PCMH that can result in addressing unmet needs of patients. However, a lack of evidence on time-varying characteristics and risk profiles of patients across PCMH models requires further research to extend the evaluation time span beyond the initial months post-diagnosis with more intensive treatment per cancer type.

The estimates of all measures in ACO models varied considerably across participating providers, and our review found a rather mixed impact on cancer care outcomes. It appears that improvements in some of the key cancer care domains, including screening, surveillance, and end-of-life care are more responsive to the ACO model than surgical outcomes in certain settings. Improvement in specialized outcome measures in cancer care may be more difficult to achieve through the ACO model, as most of the ACO quality metrics are not specifically focused on the complex care coordination in oncology practice. Additionally, the effects derived from ACO policies may become stronger over time as organizational changes take place, and the culture of coordination takes shape. As more data become available, covering the existing gap in time trends and lack of value-based metrics will be important to better evaluate the impact of the ACO model in cancer care.

The findings of this study also suggest promising cost savings from transition to prospective reimbursement models, in which financial risks and accountability are redistributed towards providers in cancer care; these results are consistent with such findings in non-cancer settings [6, 9–11]. Although there is lack of evidence on whether quality of care was compromised in alternative payment models, resource use improvements were somewhat evident in per-diem payment, pay for performance, episode-based reimbursement, and Medicare Modernization Act reforms. Despite this, the effect size of the cost savings seemed to fall short in bending the cost curve, due to the absence of more aggressive payment reforms in cancer practice. Understanding the multiple factors influencing the impact of value-based purchasing models is crucial to evaluating these types of reform that are tailored to the specific setting and target population. At a higher level, general healthcare policies and regulations as well as other quality improvement initiatives are important to the success of alternative payment models. However, the structure of the healthcare systems, provider characteristics, patient population and preferences, and their risk profiles are the key components in designing, implementing, and evaluating payment reforms, for which there was no evidence available according to our findings. In addition, the complexity of quality metrics is important to consider in evaluating the appropriateness and effectiveness of financial incentives, as providers need to understand the metrics and link them to the incentives.

Our study should be interpreted in the context of several important limitations. First, there is possibility of publication bias, as is part of the challenges associated with conducting SLRs. However, additional resources were identified through hand-searching the reference list of publications. Second, the possibility of selection bias in patients and policy interventions is inherent to the design of the included studies. Our careful attention to the quality of the evaluation research from which we captured data allowed for transparency and a full understanding of the strength of evidence using the ROBINS-I risk-assessment tool. According to our findings provided in Table 4, nearly half of the studies were subject to confounding bias, which highlights the difficulty of evaluating health policy reforms using observational data. Failure to evaluate potential confounders across the included studies may have biased our results towards erroneous conclusions on the impact of payment reforms. This observation warrants future studies that are at the very least quasi experimental designs with either interrupted time series or pre-post difference in differences construct. Furthermore, the use of manual or propensity score matching is essential to mitigate the imbalances due to selection bias.

**Table 4:** Risk of bias assessment using ROBINS-I tool for non-interventional studies.

Study (Author, year)	Risk of confounding bias	Risk of selection bias	Risk of bias in classification of interventions	Risk of bias to deviations from intended interventions	Risk of bias due to missing data	Risk of bias in measurement of outcomes	Risk of bias in reporting the results	Overall risk of bias
Colla, 2012	Serious	Moderate	Moderate	Low	Low	Moderate	Low	Moderate
Colla, 2013	Low	Low	Moderate	Low	Low	Moderate	Low	Low
Colligan, 2017	Low	Low	Moderate	Low	Low	Moderate	Low	Low
Elliott, 2010	Low	Low	Moderate	Low	Low	Moderate	Low	Low
Ems, 2018	Low	Low	Moderate	Low	Low	Moderate	Low	Low
Herrel, 2015	Serious	Moderate	Moderate	Low	Low	Moderate	Low	Moderate
Herrel, 2016	Low	Low	Moderate	Low	Low	Moderate	Low	Low
Hollenbeck, 2017	Serious	Moderate	Moderate	Low	Low	Moderate	Low	Moderate
Hoverman,2011	Serious	Moderate	Moderate	Low	Low	Moderate	Low	Moderate
Jacobson, 2010	Serious	Moderate	Moderate	Low	Low	Moderate	Low	Moderate
Kohler,2015	Serious	Low	Moderate	Low	Low	Moderate	Low	Low
Konski,2014	Moderate	Moderate	Moderate	Low	Low	Moderate	Low	Moderate
Kreys, 2013	Serious	Moderate	Moderate	Low	Low	Moderate	Low	Moderate
Kuntz, 2014	Moderate	Moderate	Low	Low	Low	Moderate	Low	Moderate
Kwon, 2018	Serious	Moderate	Moderate	Low	Low	Moderate	Low	Moderate
Lam, 2018	Low	Low	Moderate	Low	Low	Moderate	Low	Low
Mendenhal, 2018	Moderate	Moderate	Moderate	Low	Low	Moderate	Low	Moderate
Meyer, 2017	Low	Low	Moderate	Low	Low	Moderate	Low	Low
Neubauer, 2010	Serious	Moderate	Moderate	Low	Low	Moderate	Low	Moderate
Newcomer, 2014	Serious	Moderate	Moderate	Low	Low	Moderate	Low	Moderate
Schwartz, 2015	Low	Low	Moderate	Low	Low	Moderate	Low	Low
Shin, 2017	Serious	Moderate	Moderate	Low	Low	Moderate	Low	Moderate
Wang, 2017	Low	Low	Moderate	Low	Low	Moderate	Low	Low
Waters, 2019	Low	Low	Moderate	Low	Low	Moderate	Low	Low
White, 2015	Moderate	Moderate	Moderate	Low	Low	Low	Low	Low
Wright, 2018	Low	Low	Moderate	Low	Low	Moderate	Low	Low

The nature of variability across the studies, together with clinical, methodological, and statistical sources of heterogeneity were other barriers to evaluating the impact of reforms. There was no group of studies sufficiently homogeneous in terms of patients, interventions, or outcome measures to pool data or perform a meta-analysis to estimate the overall impact of each model. This likely reflects the developing nature of the policy reforms in the fast-evolving field of cancer. From a practical point of view, this study provides a tabular summary of all studies related to each key piece of information identified as important for resource use and quality improvement in cancer care. The findings from this evidence review should be useful to policymakers, providers, and payers since it covers an important aspect of cancer care reform.

## Conclusion

Of the implicit payment models driving quality improvements in cancer care, provider’s adherence to oncology pathways was significantly effective in resource use improvement. Despite this, it was difficult to get a clear picture of the effect that participating in PCMHs has on cancer care outcomes in the long term due to an insufficient number of evaluations. Much anticipated, but overdue, is the impact of ACO models with ambitions to capitalize on the cost savings of better care coordination in management of chronic diseases. However, the evaluations of ACOs in cancer care found mixed results, with Medicare Pioneer ACO demonstrating some reduction in utilization of certain low-value services in the first year. The findings also suggest promising improvement in resource utilization and cost control after transition to prospective payment models, but, further primary research is needed to apply robust measures of performance and quality to better ensure that providers are delivering high-value, quality care to their patients, while reducing the cost of care.

## Supporting information

S1 Table Search strategy in Embase. (DOCX)

S2 Table Search strategy in PubMed. (DOCX)

S3 Table Search strategy in Cochrane. (DOCX)

S4 Table Definition of the payment reforms and associated delivery models included in the SLR. (DOCX)

S5 Table Prisma checklist. (DOCX)
