# Supplementary material for: The impact of provider payment reforms and associated care delivery models on cost and quality in cancer care: A systematic literature review
Source: PLoS One. 2019 Apr 5;14(4):e0214382. doi: 10.1371/journal.pone.0214382 (PMC6450626; doi:10.1371/journal.pone.0214382)
Supplement: S1 Table — (DOCX) [file pone.0214382.s001.docx]

S1 Table. Search Strategy in Embase.

| Search criteria | Search terms | Hits |
| --- | --- | --- |
| **Population** | |  |
| 1 | ‘Cancer’ OR ‘Cancer care’:ab,ti OR ‘Cancer services’:ab,ti OR ‘Oncology practice’:ab,ti OR ‘Oncology Services’:ab,ti | [3,799,851](http://www.embase.com/) |
| **Interventions: care delivery and payment models** | |  |
| 2 | ‘Payment model’:ab,ti OR ‘reimbursement methods’:ab,ti OR ‘payment methods’:ab,ti OR ‘payment reform’:ab,ti OR ‘Fee for service’:ab,ti OR ‘Fe- fo- service’:ab,ti OR ‘FFS’:ab,ti OR ‘PCMHs’:ab,ti OR ‘Patient-centred oncology medical home’:ab,ti OR ‘oncology medical home’:ab,ti OR ‘Bundled payments’:ab,ti OR ‘ACOs’:ab,ti OR ‘Accountable care organizations’:ab,ti OR ‘Oncology Care Model’:ab,ti OR ‘OCM’:ab,ti OR ‘Value-based payment’:ab,ti OR ‘pay for performance’:ab,ti OR ‘P4P’:ab,ti OR ‘Capitation’:ab,ti OR ‘Global budget’:ab,ti OR ‘financial risk-sharing’ OR ‘clinical pathway adoption‘:ab,ti OR ‘oncology pathway adoption‘:ab,ti OR ‘pathway adoption‘:ab,ti | [17,577](http://www.embase.com/) |
| **Outcomes** | |  |
| 3 | ‘Chemotherapy medications’:ab,ti OR ‘bundle prices’:ab,ti OR ‘Cancer care costs’:ab,ti OR ‘spending’:ab,ti OR ‘Out of pocket’:ab,ti OR ‘treatment cost’:ab,ti OR ‘cost of treatment’:ab,ti OR ‘catastrophic costs’:ab,ti OR ‘oncology spending’:ab,ti OR ‘cost’ OR ‘costs’ OR ‘budget’ OR ‘expenditure’ OR ‘Health care resource use’ OR ‘HCRU’ OR ‘resource utilization’ OR ‘quality’ OR ‘quality of care’ OR ‘health outcomes’:ab,ti OR ‘mortality’:ab,ti OR ‘survival’:ab,ti OR ‘response to treatment’:ab,ti OR ‘patient satisfaction’:ab,ti OR ‘physician visits’:ab,ti OR ‘outpatient visits’:ab,ti OR ‘ICU admissions’:ab,ti OR ‘Emergency department visits’:ab,ti OR ‘ED visits’:ab,ti OR ‘Specialist visit’:ab,ti OR ‘length of stay’:ab,ti OR ‘adherence to standard of care’:ab,ti | [4,437,276](http://www.embase.com/) |
| **Limits** | |  |
| 4 | #1 AND #2 | [2,322](http://www.embase.com/) |
| 5 | #3 AND #4 | [1,821](http://www.embase.com/) |
| 6 | #5 AND [english]/lim AND [2007-2019]/py | [1,518](http://www.embase.com/) |
