# Supplementary material for: The impact of provider payment reforms and associated care delivery models on cost and quality in cancer care: A systematic literature review
Source: PLoS One. 2019 Apr 5;14(4):e0214382. doi: 10.1371/journal.pone.0214382 (PMC6450626; doi:10.1371/journal.pone.0214382)
Supplement: S2 Table — (DOCX) [file pone.0214382.s002.docx]

S2 Table. Search Strategy in PubMed.

| Search criteria | Search terms | Hits |
| --- | --- | --- |
| **Population** | |  |
| 1 | “cancer” OR “cancer Care” [tiab] OR “cancer services” [tiab] OR “oncology” [tiab] OR “oncology Services” [tiab] | [2,105,539](https://www.ncbi.nlm.nih.gov/pubmed/?cmd=HistorySearch&querykey=1) |
| **Interventions: care delivery and payment models** | |  |
| 2 | “Payment model” [tiab] OR “Payment methods” [tiab] OR “reimbursement methods” [tiab] OR “Fee for service” [tiab] OR “fee-for-service” [tiab] OR “FFS” [tiab] OR “PCMHs” [tiab] OR “Patient-centered oncology medical home” [tiab] OR “oncology medical home” [tiab] OR “Bundled payments” [tiab] OR “ACOs” [tiab] OR “Accountable care organizations” [tiab] OR “Oncology Care Model” [tiab] OR “OCM” [tiab] OR “Value-based payment” [tiab] OR “pay for performance” [tiab] OR “P4P” [tiab] OR “Capitation” [tiab] OR “Global budget” [tiab] OR “Financial risk-sharing” [tiab] OR “Financing” [tiab] OR ”clinical pathways” [tiab] OR ”clinical guidelines” [tiab] OR “clinical pathway adoption” [tiab] OR “oncology pathway adoption”[tiab] OR “pathway adoption” [tiab] | [38,381](https://www.ncbi.nlm.nih.gov/pubmed/?cmd=HistorySearch&querykey=3) |
| **Outcomes** | |  |
| 3 | “Chemotherapy medications”[tiab] OR “bundle prices”[tiab] OR “Cancer care costs”[tiab] OR “spending”[tiab] OR “Out of pocket”[tiab] OR “treatment cost”[tiab] OR “cost of treatment”[tiab] OR “catastrophic costs”[tiab] OR “oncology spending”[tiab] OR “cost” OR “costs” OR “budget” OR “expenditure” OR “Health care resource use” OR “HCRU” OR “resource utilization” OR “quality” OR “quality of care” OR “health outcomes”[tiab] OR “mortality”[tiab] OR “survival”[tiab] OR “response to treatment”[tiab] OR “patient satisfaction”[tiab] OR “physician visits”[tiab] OR “outpatient visits”[tiab] OR “ICU admissions”[tiab] OR “Emergency department visits”[tiab] OR “ED visits”[tiab] OR “Specialist visit”[tiab] OR “length of stay”[tiab] OR “adherence to standard of care”[tiab] OR “adherence”[tiab] | 3,056,027 |
| **Limits** | |  |
| 4 | #1 AND #2 | [3,177](https://www.ncbi.nlm.nih.gov/pubmed/?cmd=HistorySearch&querykey=5) |
| 5 | #3 AND #4 | [1,987](https://www.ncbi.nlm.nih.gov/pubmed/?cmd=HistorySearch&querykey=6) |
| 6 | #5 AND Filters: Abstract; Publication date from 2007/01/01 to 2019/01/15 | [1,516](https://www.ncbi.nlm.nih.gov/pubmed/?cmd=HistorySearch&querykey=15) |
