# Supplementary material for: The impact of provider payment reforms and associated care delivery models on cost and quality in cancer care: A systematic literature review
Source: PLoS One. 2019 Apr 5;14(4):e0214382. doi: 10.1371/journal.pone.0214382 (PMC6450626; doi:10.1371/journal.pone.0214382)
Supplement: S3 Table — (DOCX) [file pone.0214382.s003.docx]

S3 Table. Search Strategy in Cochrane Library.

| Search criteria | Search terms | Hits |
| --- | --- | --- |
| **Population** | |  |
| 1 | Cancer OR cancer care OR cancer services OR oncology OR oncology services | 143,385 |
| **Interventions: care delivery and payment models** | |  |
| 2 | Payment model OR Payment methods OR reimbursement methods OR Fee for service OR fee-for-service OR FFS OR PCMH OR Patient-centered oncology medical home OR oncology medical home OR Bundled payments OR ACOs OR Accountable care organizations OR Oncology Care Model OR OCM OR Value-based payment OR pay for performance OR P4P OR Capitation OR Global budget OR Financial risk-sharing OR Financing OR clinical pathways OR clinical guidelines OR clinical pathway adoption OR oncology pathway adoption OR pathway adoption | 27,889 |
| **Outcomes** | |  |
| 3 | Chemotherapy medications OR bundle prices OR Cancer care costs OR spending OR Out of pocket OR treatment cost OR cost of treatment OR catastrophic costs OR oncology spending OR cost OR costs OR budget OR expenditure OR Health care resource use OR HCRU OR resource utilization OR quality OR quality of care OR health outcomes OR mortality OR survival OR response to treatment OR patient satisfaction OR physician visits OR outpatient visits OR ICU admissions OR Emergency department visits OR ED visits OR Specialist visit OR length of stay OR adherence to standard of care OR adherence | 369,188 |
| **Limits** | |  |
| 4 | #1 AND #2 | 5,799 |
| 5 | #3 AND #4 | 4,711 |
| 6 | #5 AND Cochrane reviews | 1,479 |
