# Supplementary material for: The impact of provider payment reforms and associated care delivery models on cost and quality in cancer care: A systematic literature review
Source: PLoS One. 2019 Apr 5;14(4):e0214382. doi: 10.1371/journal.pone.0214382 (PMC6450626; doi:10.1371/journal.pone.0214382)
Supplement: S4 Table — (DOCX) [file pone.0214382.s004.docx]

S4 Table. Category of Payment Reforms Included in the SLR

|  | Type of reform | Definition |
| --- | --- | --- |
| **Explicit payment reforms directly involving financial incentives** | Fee-for-service (FFS) | Payers reimburse providers for each service rendered to consumers. |
|  | Global payments (capitation) | Payers offer providers a fixed amount per member, often either per month or per year, regardless of service utilization. |
|  | Per-diem payment | Payers reimburse based on the number of days the patient directly receives treatment from the health care provider. |
|  | Pay for performance (P4P) | Comprises payment models that attach financial incentives/disincentives to provider performance as it ties reimbursement to metric-driven outcomes |
|  | Episode-based payment | Payers reimburse providers on the basis of expected costs for clinically-defined episodes/bundles of care |
|  | Other reimbursement reforms in oncology practice | Changes in payment policies in the Medicare Modernization Act  Endometrial cancer (EC) alternative payment (ECAP) model: a value-based healthcare reform initiated based on the Physician Payment Reform Taskforce (PPRTF) in the Society of Gynecologic Oncology (SGO) |
| **Implicit payment reforms through care coordination and delivery models** | Clinical pathway adoption | A set of clinical recommendations defining what types of services or procedures should be delivered to or ordered for specific patients. |
|  | Patient-centered medical home (PCMH) | Practices support the attainment of optimal, patient-centered outcomes through a team-based healthcare delivery with robust partnership between physicians, patients, and the patient’s family. |
|  | Accountable care organization (ACO) | Practices with a group of providers organized in a way that enables them to take accountability for the overall quality and cost of all or most of the healthcare services needed by a group of patients over a period of time. |
|  | Other major delivery reforms under the umbrella of payment reforms in cancer care | OCM: A five-year model implemented by center for Medicare and Medicaid services (CMS) to test innovative payment models that promote quality and value in chemotherapy related care |
